# Do Social Support and Strain Mediate the Relationship Between Childhood Exposures and Frailty in Later Life?

**DOI:** 10.1093/geroni/igaa057.1606

**Published:** 2020-12-16

**Authors:** Monica Williams-Farrelly, Kenneth Ferraro

**Affiliations:** Purdue University, West Lafayette, Indiana, United States

## Abstract

Previous studies have identified the early origins of physical frailty, notably poor childhood health and socioeconomic status, but relatively few studies examine whether social support in later life mitigates the influence of early noxious exposures on frailty. Given the established relationship between health and social relationships in older adults, this research uses data from the Health and Retirement study (2004-2016) to examine whether social support and strain mediate the effect of childhood exposures on frailty in later life. A series of linear regression and pathway models were estimated to test whether childhood exposures, including socioeconomic status, infectious and chronic diseases, impairments, and risky adolescent parental behaviors, were associated with phenotypic frailty (Fried et al. 2001). After adjusting for demographic and adult factors, accumulated childhood misfortune was directly (b=0.015, p<.01) and indirectly (b=0.007, p<.001) associated with more frailty. Average social support, but not strain, from one’s spouse, children, family and friends significantly mediated the relationship between accumulated misfortune and frailty (b= -.002, p<.01). Path analysis revealed that social support reduces later life frailty directly (b=-0.106 ,p<.001) and indirectly through a reduction in adult morbidity (b=-0.031, p<.001). However, counterintuitively we found that accumulated misfortune was associated with more social support. Supplemental analyses reveal that one or more infectious diseases in childhood were responsible for the positive relationship (b= 0.393, p<.001). These results have implications for how we may reduce the burden of frailty on those who have experienced misfortune early in life.

